# 4,4′-[Butane-1,4-diylbis(nitrilo­methyl­idyne)]dibenzonitrile

**DOI:** 10.1107/S160053680802744X

**Published:** 2008-08-30

**Authors:** Hoong-Kun Fun, Reza Kia, Hadi Kargar

**Affiliations:** a X-ray Crystallography Unit, School of Physics, Universiti Sains Malaysia, 11800 USM, Penang, Malaysia; b Department of Chemistry, School of Science, Payame Noor University (PNU), Ardakan, Yazd, Iran

## Abstract

The title Schiff base compound, C_20_H_18_N_4_, lies across a crystallographic inversion centre and adopts *E* configurations with respect to the C=N bonds. The asymmetric unit of the compound is composed of one half-mol­ecule. The imino group is coplanar with the benzene ring. Within the mol­ecule, the planar units are parallel but extend in opposite directions from the methyl­ene bridge. In the crystal structure, neighbouring mol­ecules are linked together by weak inter­molecular C—H⋯N hydrogen bonds involving the cyano N atoms. These form ten-membered rings, generating *R* ^2^_2_(10) ring motifs, and link the mol­ecules along the *c* axis.

## Related literature

For bond-length data, see: Allen *et al.* (1987 ▶). For hydrogen-bond motifs, see: Bernstein *et al.* (1995 ▶). For information on Schiff base ligands, their complexes and applications, see, for example: Fun, Kargar & Kia (2008 ▶); Fun, Kia & Kargar (2008 ▶); Fun & Kia (2008*a* ▶,*b* ▶); Calligaris & Randaccio (1987 ▶); Casellato & Vigato (1977 ▶).
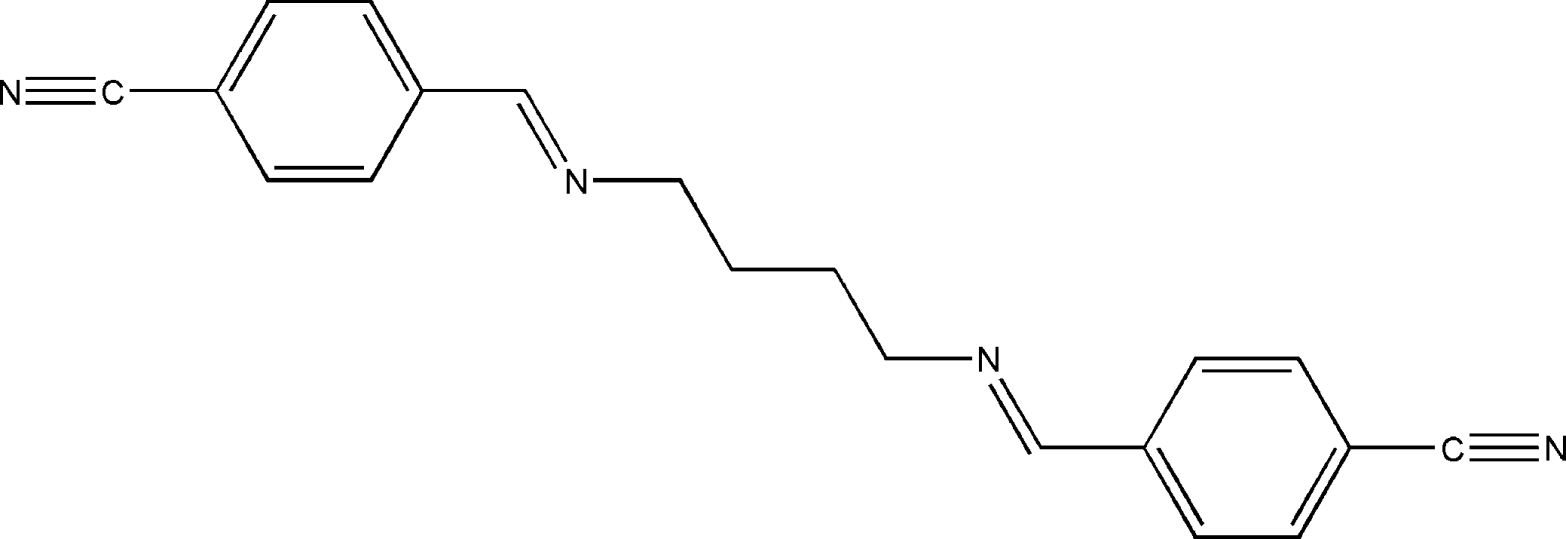


## Experimental

### 

#### Crystal data

C_20_H_18_N_4_
*M* *_r_* = 314.38
Monoclinic, 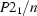

*a* = 4.9720 (2) Å
*b* = 10.5047 (5) Å
*c* = 16.0315 (6) Å
β = 97.220 (3)°
*V* = 830.68 (6) Å^3^
*Z* = 2
Mo *K*α radiation
μ = 0.08 mm^−1^
*T* = 100.0 (1) K
0.52 × 0.33 × 0.13 mm

#### Data collection

Bruker SMART APEXII CCD area-detector diffractometer
Absorption correction: multi-scan (**SADABS**; Bruker, 2005 ▶) *T*_min_ = 0.942, *T*_max_ = 0.990
10382 measured reflections
2603 independent reflections
2035 reflections with *I* > 2σ(*I*)
*R*_int_ = 0.027

#### Refinement

*R*[*F* ^2^ > 2σ(*F* ^2^)] = 0.046
*wR*(*F* ^2^) = 0.143
*S* = 1.11
2603 reflections
145 parameters
All H-atom parameters refined
Δρ_max_ = 0.31 e Å^−3^
Δρ_min_ = −0.20 e Å^−3^

### 

Data collection: *APEX2* (Bruker, 2005 ▶); cell refinement: *APEX2*; data reduction: *SAINT* (Bruker, 2005 ▶); program(s) used to solve structure: *SHELXTL* (Sheldrick, 2008 ▶); program(s) used to refine structure: *SHELXTL*; molecular graphics: *SHELXTL*; software used to prepare material for publication: *SHELXTL* and *PLATON* (Spek, 2003 ▶).

## Supplementary Material

Crystal structure: contains datablocks global, I. DOI: 10.1107/S160053680802744X/sj2534sup1.cif

Structure factors: contains datablocks I. DOI: 10.1107/S160053680802744X/sj2534Isup2.hkl

Additional supplementary materials:  crystallographic information; 3D view; checkCIF report

## Figures and Tables

**Table 1:** Hydrogen-bond geometry (Å, °)

*D*—H⋯*A*	*D*—H	H⋯*A*	*D*⋯*A*	*D*—H⋯*A*
C2—H2⋯N2^i^	0.945 (13)	2.541 (14)	3.3973 (14)	150.8 (12)

Symmetry code: (i) 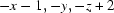.

**Fig. 1.**
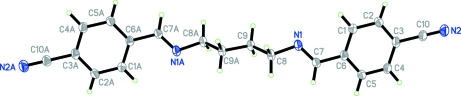
The molecular structure of (I) with atom labels and 50% probability ellipsoids for non-H atoms. The suffix A corresponds to symmetry code (-x + 1, -y, -z + 1).

**Fig. 2.**
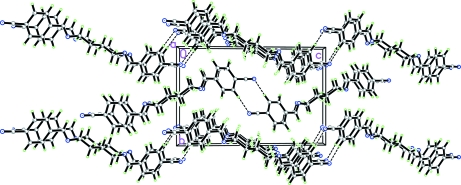
The crystal packing of (I), viewed down the a axis showing chains along the c-axis. Intermolecular interactions are shown as dashed lines.

## supplementary crystallographic information

### Comment

The condensation of primary amines with carbonyl compounds yields Schiff base compounds (Casellato & Vigato, 1977); these are still one of the most prevalent mixed-donor ligands in coordination chemistry. In the past two decades, the synthesis, structure and properties of Schiff base complexes have stimulated much interest due to their noteworthy contributions in single molecule-based magnetism, materials science and the catalysis of many reactions such as carbonylation, hydroformylation, reduction, oxidation, epoxidation and hydrolysis (Casellato & Vigato 1977). However, only a relatively small number of free Schiff base ligands have been characterized (Calligaris & Randaccio, 1987). As an extension of our work (Fun, Kargar & Kia 2008; Fun, Kia & Kargar 2008; Fun & Kia 2008*a,b*) on the structural characterization of Schiff base ligands, the structure of the title compound, (I), is reported here.

The molecule of the title compound (I, Fig 1), lies across a crystallographic inversion centre and adopts *E* configurations with respect to the C═N bonds. The bond lengths and angles are within normal ranges (Allen *et al.*,1987). The asymmetric unit of the compound is composed of one-half of the molecule. The imino group is coplanar with the benzene ring. Within the molecule, the planar units are parallel but extend in opposite directions from the methylene bridge. In the crystal structure, neighbouring molecules are linked together by weak intermolecular C—H···N hydrogen bonds involving the cyano N atoms. These form ten-membered rings, generate *R*^2^_2_(10) ring motifs (Bernstein *et al.* 1995) and link the molecules along the *c*-axis.

### Experimental

The synthetic method has been described earlier (Fun, Kia & Kargar *et al.*, 2008). Single crystals suitable for *X*-ray diffraction were obtained by evaporation of an ethanol solution at room temperature.

### Refinement

All of the hydrogen atoms were located from the difference Fourier map and refined freely with fixed isotropic displacement parameters.

### Crystal data

**Table:**

C_20_H_18_N_4_	*F*_000_ = 332
*M**_r_* = 314.38	*D*_x_ = 1.257 Mg m^−^^3^
Monoclinic, *P*2_1_/*n*	Mo *K*α radiation λ = 0.71073 Å
Hall symbol: -P 2yn	Cell parameters from 2704 reflections
*a* = 4.9720 (2) Å	θ = 3.2–30.8º
*b* = 10.5047 (5) Å	µ = 0.08 mm^−^^1^
*c* = 16.0315 (6) Å	*T* = 100.0 (1) K
β = 97.220 (3)º	Block, colourless
*V* = 830.68 (6) Å^3^	0.52 × 0.33 × 0.13 mm
*Z* = 2	

### Data collection

**Table:**

Bruker SMART APEXII CCD area-detector diffractometer	2603 independent reflections
Radiation source: fine-focus sealed tube	2035 reflections with *I* > 2σ(*I*)
Monochromator: graphite	*R*_int_ = 0.027
*T* = 100.0(1) K	θ_max_ = 30.9º
φ and ω scans	θ_min_ = 2.3º
Absorption correction: multi-scan(*SADABS*; Bruker, 2005)	*h* = −7→7
*T*_min_ = 0.942, *T*_max_ = 0.990	*k* = −12→15
10382 measured reflections	*l* = −23→20

### Refinement

**Table:**

Refinement on *F*^2^	Secondary atom site location: difference Fourier map
Least-squares matrix: full	Hydrogen site location: inferred from neighbouring sites
*R*[*F*^2^ > 2σ(*F*^2^)] = 0.046	All H-atom parameters refined
*wR*(*F*^2^) = 0.143	*w* = 1/[σ^2^(*F*_o_^2^) + (0.08*P*)^2^ + 0.042*P*] where *P* = (*F*_o_^2^ + 2*F*_c_^2^)/3
*S* = 1.12	(Δ/σ)_max_ = 0.001
2603 reflections	Δρ_max_ = 0.31 e Å^−^^3^
145 parameters	Δρ_min_ = −0.20 e Å^−^^3^
Primary atom site location: structure-invariant direct methods	Extinction correction: none

### Special details

**Table:**

Experimental. The low-temperature data was collected with the Oxford Cyrosystem Cobra low-temperature attachment.
Geometry. All esds (except the esd in the dihedral angle between two l.s. planes) are estimated using the full covariance matrix. The cell esds are taken into account individually in the estimation of esds in distances, angles and torsion angles; correlations between esds in cell parameters are only used when they are defined by crystal symmetry. An approximate (isotropic) treatment of cell esds is used for estimating esds involving l.s. planes.
Refinement. Refinement of F^2^ against ALL reflections. The weighted R-factor wR and goodness of fit S are based on F^2^, conventional R-factors R are based on F, with F set to zero for negative F^2^. The threshold expression of F^2^ > 2sigma(F^2^) is used only for calculating R-factors(gt) etc. and is not relevant to the choice of reflections for refinement. R-factors based on F^2^ are statistically about twice as large as those based on F, and R- factors based on ALL data will be even larger.

### Fractional atomic coordinates and isotropic or equivalent isotropic displacement parameters (Å^2^)

**Table:**

	*x*	*y*	*z*	*U*_iso_*/*U*_eq_	
N1	0.31548 (17)	0.07369 (8)	0.67193 (5)	0.0242 (2)	
N2	−0.5866 (2)	0.17550 (9)	1.01544 (6)	0.0364 (3)	
C1	0.0130 (2)	0.06350 (9)	0.81305 (6)	0.0255 (2)	
C2	−0.1490 (2)	0.06318 (10)	0.87727 (6)	0.0269 (2)	
C3	−0.3002 (2)	0.17138 (9)	0.89127 (6)	0.0240 (2)	
C4	−0.2917 (2)	0.27859 (10)	0.84069 (6)	0.0260 (2)	
C5	−0.1254 (2)	0.27847 (10)	0.77740 (6)	0.0246 (2)	
C6	0.02661 (18)	0.17141 (9)	0.76284 (6)	0.0213 (2)	
C7	0.20222 (19)	0.17330 (9)	0.69517 (6)	0.0218 (2)	
C8	0.49150 (19)	0.08750 (10)	0.60619 (6)	0.0245 (2)	
C9	0.39698 (18)	0.00237 (9)	0.53129 (6)	0.0226 (2)	
C10	−0.4614 (2)	0.17317 (10)	0.95980 (6)	0.0276 (2)	
H1	0.121 (3)	−0.0110 (13)	0.8041 (8)	0.033 (3)*	
H2	−0.153 (3)	−0.0091 (13)	0.9122 (8)	0.035 (3)*	
H4	−0.401 (3)	0.3560 (13)	0.8497 (8)	0.032 (3)*	
H5	−0.116 (2)	0.3552 (12)	0.7446 (8)	0.033 (3)*	
H7	0.224 (2)	0.2575 (12)	0.6706 (8)	0.028 (3)*	
H8A	0.502 (2)	0.1786 (11)	0.5893 (8)	0.028 (3)*	
H8B	0.678 (2)	0.0626 (11)	0.6313 (7)	0.025 (3)*	
H9A	0.222 (2)	0.0346 (11)	0.5017 (7)	0.024 (3)*	
H9B	0.363 (2)	−0.0864 (12)	0.5502 (8)	0.029 (3)*	

### Atomic displacement parameters (Å^2^)

**Table:**

	*U*^11^	*U*^22^	*U*^33^	*U*^12^	*U*^13^	*U*^23^
N1	0.0259 (4)	0.0289 (4)	0.0189 (4)	−0.0028 (3)	0.0071 (3)	−0.0027 (3)
N2	0.0469 (6)	0.0336 (5)	0.0326 (5)	−0.0050 (4)	0.0199 (4)	−0.0056 (4)
C1	0.0297 (5)	0.0240 (5)	0.0242 (5)	0.0005 (4)	0.0086 (4)	−0.0012 (4)
C2	0.0333 (5)	0.0267 (5)	0.0225 (5)	−0.0032 (4)	0.0102 (4)	0.0004 (4)
C3	0.0246 (5)	0.0285 (5)	0.0199 (5)	−0.0065 (4)	0.0067 (3)	−0.0060 (3)
C4	0.0277 (5)	0.0265 (5)	0.0248 (5)	−0.0017 (4)	0.0079 (4)	−0.0046 (4)
C5	0.0287 (5)	0.0241 (5)	0.0221 (5)	−0.0022 (4)	0.0073 (4)	−0.0009 (3)
C6	0.0213 (4)	0.0247 (5)	0.0183 (4)	−0.0041 (3)	0.0041 (3)	−0.0040 (3)
C7	0.0231 (4)	0.0248 (5)	0.0182 (4)	−0.0047 (3)	0.0045 (3)	−0.0018 (3)
C8	0.0229 (5)	0.0321 (5)	0.0200 (5)	−0.0042 (4)	0.0080 (3)	−0.0036 (4)
C9	0.0189 (4)	0.0307 (5)	0.0189 (4)	−0.0028 (4)	0.0052 (3)	−0.0036 (4)
C10	0.0325 (5)	0.0267 (5)	0.0252 (5)	−0.0055 (4)	0.0104 (4)	−0.0054 (4)

### Geometric parameters (Å, °)

**Table:**

N1—C7	1.2663 (13)	C4—H4	0.999 (13)
N1—C8	1.4594 (12)	C5—C6	1.3910 (14)
N2—C10	1.1505 (13)	C5—H5	0.967 (13)
C1—C2	1.3845 (14)	C6—C7	1.4757 (13)
C1—C6	1.3969 (14)	C7—H7	0.979 (13)
C1—H1	0.971 (13)	C8—C9	1.5233 (13)
C2—C3	1.3963 (15)	C8—H8A	0.998 (12)
C2—H2	0.946 (14)	C8—H8B	0.996 (12)
C3—C4	1.3916 (14)	C9—C9^i^	1.5220 (18)
C3—C10	1.4392 (14)	C9—H9A	0.997 (11)
C4—C5	1.3869 (14)	C9—H9B	1.002 (12)
			
C7—N1—C8	117.36 (8)	C1—C6—C7	120.70 (8)
C2—C1—C6	120.36 (9)	N1—C7—C6	122.09 (9)
C2—C1—H1	119.5 (7)	N1—C7—H7	123.5 (7)
C6—C1—H1	120.1 (7)	C6—C7—H7	114.4 (7)
C1—C2—C3	119.52 (9)	N1—C8—C9	110.94 (8)
C1—C2—H2	120.1 (8)	N1—C8—H8A	110.5 (7)
C3—C2—H2	120.3 (8)	C9—C8—H8A	111.8 (7)
C4—C3—C2	120.58 (9)	N1—C8—H8B	107.0 (7)
C4—C3—C10	119.66 (9)	C9—C8—H8B	110.1 (7)
C2—C3—C10	119.75 (9)	H8A—C8—H8B	106.4 (10)
C5—C4—C3	119.35 (9)	C9^i^—C9—C8	111.85 (9)
C5—C4—H4	119.5 (7)	C9^i^—C9—H9A	108.5 (6)
C3—C4—H4	121.2 (7)	C8—C9—H9A	109.9 (7)
C4—C5—C6	120.66 (9)	C9^i^—C9—H9B	108.8 (7)
C4—C5—H5	118.1 (8)	C8—C9—H9B	110.8 (7)
C6—C5—H5	121.2 (8)	H9A—C9—H9B	106.8 (10)
C5—C6—C1	119.52 (9)	N2—C10—C3	178.83 (11)
C5—C6—C7	119.78 (8)		
			
C6—C1—C2—C3	0.35 (15)	C2—C1—C6—C7	178.98 (9)
C1—C2—C3—C4	0.77 (15)	C8—N1—C7—C6	−178.16 (8)
C1—C2—C3—C10	−177.82 (9)	C5—C6—C7—N1	−170.25 (9)
C2—C3—C4—C5	−1.76 (15)	C1—C6—C7—N1	10.32 (14)
C10—C3—C4—C5	176.82 (9)	C7—N1—C8—C9	−122.71 (9)
C3—C4—C5—C6	1.66 (14)	N1—C8—C9—C9^i^	−170.18 (10)
C4—C5—C6—C1	−0.57 (15)	C4—C3—C10—N2	−87 (6)
C4—C5—C6—C7	180.00 (8)	C2—C3—C10—N2	92 (6)
C2—C1—C6—C5	−0.45 (15)		

Symmetry codes: (i) −*x*+1, −*y*, −*z*+1.

### Hydrogen-bond geometry (Å, °)

**Table:**

*D*—H···*A*	*D*—H	H···*A*	*D*···*A*	*D*—H···*A*
C2—H2···N2^ii^	0.945 (13)	2.541 (14)	3.3973 (14)	150.8 (12)

Symmetry codes: (ii) −*x*−1, −*y*, −*z*+2.
